# Expression of Caspase-1 variants induced ER stress

**DOI:** 10.1186/1546-0096-13-S1-P17

**Published:** 2015-09-28

**Authors:** H Luksch, V Schlipfenbacher, S Köhler, F Münch, S Winkler, F Schulze, J Roesler, A Rösen-Wolff

**Affiliations:** 1 University Hospital Carl Gustav Carus, Department of Pediatrics, Dresden, Germany

## Introduction

Caspase-1 is a proinflammatory enzyme that is activated by the NLRP3 inflammasome in response to endoplasmic reticulum (ER) stress independent of the classical unfolded protein response. This finding linked ER stress to chronic inflammatory diseases. In patients suffering from unexplained recurrent febrile episodes we detected several genetic variants of *CASP1* leading to reduced enzymatic activity due to destabilization of the caspase-1 dimer interface.

## Objective

We investigated a possible association of expression level and reduced enzymatic activity of variant caspase-1 with impaired ER-stress responses.

## Methods

We analysed ER stress markers in THP-1 cells and lymphoblastoid cells lines (LCL, EBV transformed B cells) from individuals harbouring *CASP1* variants and healthy donors. Additionally, we knocked-down endogenous caspase-1 in THP-1 cells and in LCL to determine caspase-1 involvement in ER stress responses. Knock-down of caspase-1 was functionally verified by FLICA-assay. We used Western blot analyses and quantitative real time RT-PCR to examine mRNA expression of genes involved in ER stress. In a next step, we infected LCL with microorganism, such as *Salmonella typhimurium*, to investigate the inflammation dependent cell death regulated by the proteolytic activity of caspase-1.

## Results

Activation of NLRP3 inflammasome and also induction of ER stress by tunicamycin lead to secretion of IL-1β, IL-8, and TNF-α. In an analogous manner, LPS induced activation of the NLRP3 inflammasome or treatment of cells with tunicamycin resulted in an increased expression of ER stress related genes in native THP-1 cells. Expression of ER stress marker genes was also increased in native patients’ LCLs with reduced enzymatic activity of caspase-1. After infection with microorganisms the amount of death cell was significantly increased in LCL expressing wildtype procaspase-1 compared to LCL with variant procaspase-1. In patients’ LCLs this cell death could be prevented more efficiently by YVAD (caspase-1 inhibitor) and zVAD (general caspase inhibitor) than in wildtype LCLs. Cell death during inflammation was dependent on enzymatic activity of caspase-1.

## Conclusion

This data indicate that ER stress induces activation of wildtype caspase-1 and that vice versa inflammasome activation induces ER stress in different cell types. In LCL of patients harbouring caspase-1 variants, knock-down of pro-caspase-1 reduces ER stress. This indicates a role of variant caspase-1 in dealing with intrinsic danger signals.

This study was supported by the German Research Foundation (DFG, KFO 249) and by a MeDDrive project (University of Technology, Medical Faculty) to HL.

